# Supplementary material for: Detection of Hypertension-Induced Changes in Erythrocytes by SERS Nanosensors
Source: Biosensors (Basel). 2022 Jan 8;12(1):32. doi: 10.3390/bios12010032 (PMC8773528; doi:10.3390/bios12010032)
Supplement: Supplementary file 1 [file biosensors-12-00032-s001.zip › biosensors-1498627-supplementary.pdf]

biosensors

Supporting Information

Detection of Hypertension-Induced Changes in Erythrocytes by SERS Nanosensors

Evelina I. Nikelshparg [1,*], Adil A. Baizhumanov [1], Zhanna V. Bochkova [1], Sergey M. Novikov [2], Dmitry I. Yakubovsky [2], Aleksey V. Arsenin [2], Valentyn S. Volkov [2], Eugene A. Goodilin [3,4], Anna A. Semenova [3], Olga Sosnovtseva [5], Georgy V. Maksimov [1,6] and Nadezda A. Brazhe [1,*]

The traditional method used to calculate enhancement factor (EF) is to compare the chosen peak intensity in the Raman and SERS spectra taking into the account concentration of a molecule that was used in the SERS experiment and in "traditional" Raman measurements:

$$EF = C_{RS}/C_{SERS} \times I_{SERS}/I_{RS} \quad (1)$$

where C_{RS} and C_{SERS} are concentrations of analyte molecules in Raman and SERS measurements, respectively, and I_{SERS} and I_{RS} are the intensities of a chosen peak in SERS and Raman spectra, respectively [1].

Here, we calculated EF for the peak at 1638 cm^{-1} corresponding to the vibrations of methine bridges in the heme molecule. Figure S1b demonstrates the SERS and Raman spectra of erythrocyte samples obtained by blood dilutions carried out 2000 times (SERS experiment) and 50 times (Raman experiment). The registration time, laser power, objective, etc., were the same. The concentration of Hb molecules giving Raman scattering is equal to the overall concentration of Hb in a sample used for Raman spectra measurements. Here, blood dilution was carried out 50 times and the Hb concentration in the blood is appr. 10 mM; thus, the Hb concentration in the sample was 0.2 mM = 2×10^{-4} M. In the SERS experiment, blood dilution was carried out 2000 times. Moreover, only membrane-bound Hb molecules contribute to SERS spectra and the amount of Hb_{mb} is appr. 0.5% out the whole Hb in erythrocytes [2]. Thus, the concentration of Hb_{sm} in the sample used to record SERS spectra was 2.5×10^{-8} M. However, not all Hb_{mb} molecules give a SERS signal, only those located in the submembrane region of erythrocyte from its side in contact with AgNSS (see Figure 1). Thus, the concentration of Hb_{mb} giving SERS spectra is at least two times less: app. 10^{-8} M. Therefore, the EF for the peak at 1638 cm^{-1} is:

$$EF_{1638} \approx [2 \times 10^{-4}/10^{-8}] \times [240/190] = 2.5 \times 10^{4} \quad (2)$$

We cannot calculate the EF for erythrocyte ghosts, since it impossible to record the Raman scattering of this preparation without AgNSS due to the very low amount ghosts that can be obtained from the blood.

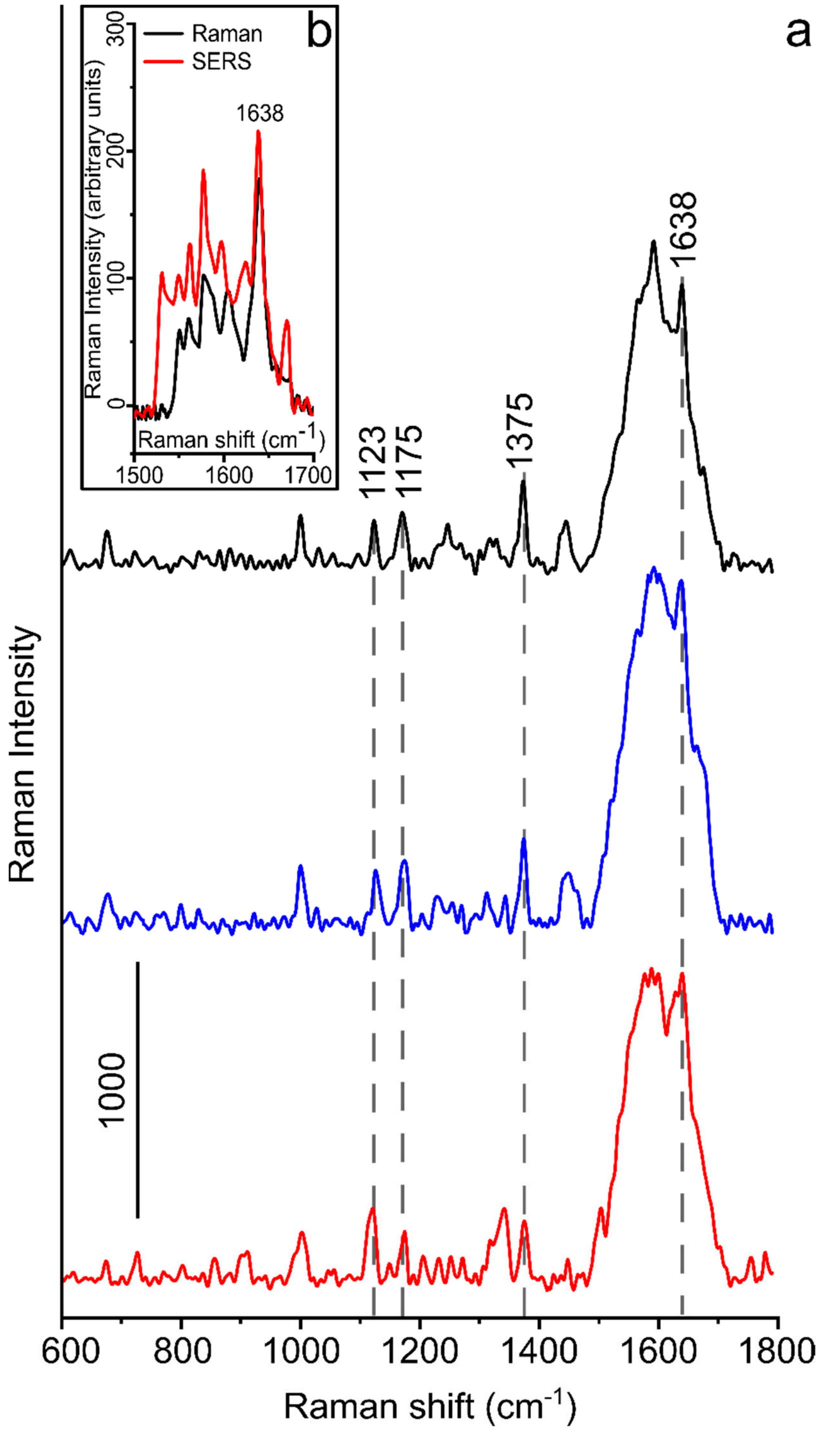

Figure S1. (**a**) SERS spectra of erythrocyte ghosts recorded from different places in a randomly chosen AgNSS. X-axis, Raman shift, cm^{-1}; Y-axis, SERS intensity, vertical scale bar corresponds to 1000 arbitr.u. For a clearer representation, the spectra are shifted vertically. Numbers above a peak show maximum positions. The similarity of the spectral structure demonstrates the high reproducibility of the SERS spectra of erythrocyte ghosts. (**b**) Raman and SERS spectra of erythrocyte suspensions. X-axis, Raman shift, cm^{-1}; Y-axis, Raman or SERS intensity. Raman spectrum (black) was recorded from the erythrocyte suspension obtained by a 50-time blood dilution; SERS spectrum (red) was recorded from an erythrocyte sample obtained by a 2000-time blood dilution. Laser wavelength was 514 nm with a power of 1 mW per registration spot, with 60 s accumulation time; objective 20 x, NA 0.4.

References:

1. Moskovits, M. Surface-enhanced spectroscopy. *Rev. Mod. Phys.* **1985**, *57*, 783–826. https://doi.org/10.1103/RevModPhys.57.783.
2. Shaklai, N.; Yguerabide, J.; Ranney, H.M. Interaction of hemoglobin with red blood cell membranes as shown by a fluorescent chromophore. *Biochemistry* **1977**, *16*, 5585–5592. https://doi.org/10.1021/bi00644a031.
